# Genome Sequence of *Legionella anisa*, Isolated from a Respiratory Sample, Using an Amoebal Coculture Procedure

**DOI:** 10.1128/genomeA.00031-14

**Published:** 2014-02-06

**Authors:** Isabelle Pagnier, Olivier Croce, Catherine Robert, Didier Raoult, Bernard La Scola

**Affiliations:** Unité de Recherche sur les Maladies Infectieuses et Tropicales Emergentes, CNRS, UMR 6236, IRD 198, Faculté de Médecine, Aix-Marseille Université, Marseille, France

## Abstract

*Legionella anisa* is a gammaproteobacterium from the class *Legionellaceae*, which is responsible for nosocomial pneumonia. We sequenced the genome from the *L. anisa* strain Linanisette, which was recovered from a clinical sample using an amoebal coculture procedure but not with standard culture methods.

## GENOME ANNOUNCEMENT

*Legionella anisa* was first isolated from an environmental sample, in drinking and cooling-tower water (1). Some strains were also implicated in several cases of Legionnaire’s disease (2, 3). We isolated a strain of *L. anisa*, Linanisette, using an amoebal coculture procedure. Common bacterial detections and culture methods, such as those used for *Legionella* spp., did not allow its isolation (4). This is a Gram-negative and Gimenez-positive bacillus, classified in the genus *Legionella*. Regarding the *mip* gene (macrophage infectivity potentiator), the strain Linanisette exhibits 100% similarity using BLASTn (5) with the type strain of *L. anisa* (Genbank accession no. U91607). After cocultivation with the amoebic species *Acanthamoeba polyphaga*, *L. anisa* Linanisette is unable to grow on Columbia with 5% sheep blood agar and is able to grow after 3 days on buffered charcoal yeast extract (BCYE) medium, under a 2.5-to-5% CO_2_ atmosphere. This bacterium is oxidase, catalase, and gelatinase positive.

The genome was pyrosequenced with a 454 GS FLX Titanium platform (Roche, Branford, CT) (6) using paired-end and shotgun techniques. A total of 437,769 reads were assembled with Newbler software version 2.6 (Roche). Scaffolding was improved using Opera software version 1.4 (7), combined with GapFiller version 1.10 (8) and manual curation.

The draft genome of *L. anisa* Linanisette consists of 20 scaffolds of 56 contigs containing 4,319,808 bp and an estimated size, including gaps, of 4,341,831 bp. The G+C content of this genome is 37.97%, which is similar to those of the other closed *Legionella* spp. Using BLASTn, Aragorn (9), and RNAmmer (10), the draft genome was shown to contain 39 RNA genes, including 3 rRNAs in a single operon and 36 tRNAs.

Potential coding sequences (CDSs) were predicted using the Prodigal software (11). The assignment of protein functions was performed by searching against the GenBank, Clusters of Orthologous Groups, and Pfam databases using BLASTp (12–14). A total of 3,873 genes were identified, representing a coding capacity of 3,755,010 bp (86.4% of the sequenced genome). Of these genes, 164 (4.2%) were found to be putative proteins, 1,530 (39.5%) were assigned as hypothetical proteins, and 51 (1.3%) encode proteins of unknown function. Moreover, 3,845 genes match at least one sequence in the COG database (12), using BLASTp default parameters.

*L. anisa* Linanisette presents some genes annotated as encoding resistance proteins (22 genes), such as those related to copper resistance. The RAST server (15) showed that *Legionella pneumophila* strain Lens (accession no. NC_006369) is the closest fully annotated genome currently available in GenBank. A brief comparison of the metabolic functions against *L. pneumophila* Lens highlights that *L. anisa* Linanisette has more genes involved in drug resistance and oxidative stress. Moreover, *L. anisa* Linanisette presents more genes involved in conjugative transfer.

### Nucleotide sequence accession numbers.

The *L. anisa* Linanisette genome is available in DDBJ/EMBL/GenBank under the accession no. CANP01000001 to CANP01000056.
